# Determination, Validation, and Development of Prediction Scores Related to Hypoglycemia Risk Factors Among Ambulatory Type 2 Diabetes Mellitus Patients in Bali, Indonesia

**DOI:** 10.2174/0115733998375680250509052027

**Published:** 2025-05-23

**Authors:** Made Krisna Adi Jaya, Fita Rahmawati, Nanang Munif Yasin, Zullies Ikawati

**Affiliations:** 1 Faculty of Pharmacy, Universitas Gadjah Mada, Yogyakarta, Indonesia;; 2 Department of Pharmacy, Faculty of Math and Science, Universitas Udayana, Bali, Indonesia

**Keywords:** Ambulatory, hypoglycemia, focus group discussions, diabetic neuropathy, type 2 diabetes mellitus, blood glucose

## Abstract

**Introduction:**

Hypoglycemia is a complication that can disrupt the continuity of therapy for Type 2 Diabetes Mellitus (T2DM) patients. Therefore, ambulatory T2DM patients need supportive treatment to ensure the safety of their medication. Currently, health workers lack a standardized reference for assessing the risk of hypoglycemia in these patients. Therefore, this study aimed to determine, validate, and develop a prediction score of risk factors influencing hypoglycemia in T2DM patients.

**Methods:**

The psychometric properties method was used in this study design, including item development, content validity, criterion validity, and construct validity. During the process of item development, Focus Group Discussions (FGDs) with experts were used. Known predictors were then validated through panelists' content assessment. Risk factors for hypoglycemia from item development and content validation were tested for criterion and construct validation using a case-control method.

**Results and Discussion:**

Eight significant predictors caused hypoglycemia, including insulin use, SU use, insulin-SU combination use, CKD, diabetic neuropathy, uncontrolled blood glucose, >5-year DM duration, and history of severe hypoglycemia, and the total score of risk factors was 24. The categories were defined as follows: scores of 0-8 as low-risk, 9-16 as moderate-risk, and 17-24 as high-risk. There was a positive linear relationship between the total score and risk category (*p*<0.05; r^2^: 0.959).

**Conclusion:**

This risk factor and score prediction model can be used by health workers in clinical practice to predict the risk of ambulatory hypoglycemia in T2DM patients because it meets content, criterion, and construct validity.

## 1 INTRODUCTION

Hypoglycemic events remain a concern for ambulatory T2DM patients due to the increasing reported incidence rate. This condition can disrupt the productive activities of these patients, and severe hypoglycemia may lead to coma, seizures, or even death [1, 2]. Mild to severe hypoglycemia cases occurring in patients at home are difficult to detect and go unreported, making it challenging to identify such cases in ambulatory T2DM patients. Recurrent episodes of moderate to severe hypoglycemia can alter the counterregulatory response to decrease blood glucose levels and lead to hypoglycemia unawareness [3]. This condition can trigger serious hypoglycemia cases in the future. The incidence of DM in Indonesia is steadily rising. In 2015, there were 10 million reported cases, and by 2040, this number is projected to increase to 16.2 million [2, 4, 5].

In developing countries, the ratio of health workers to patients is often less than ideal [4, 6, 7]. T2DM patients, in particular, primarily receive curative care aimed at achieving therapeutic goals, while many do not receive comprehensive preventive and promotive services [8-10]. This situation makes it difficult for health workers to categorize emergency patients who need to receive supportive services, such as counseling, home care services, and self-management support training, which can be provided by pharmacists, nurses, or nutritionists to prevent hypoglycemia and accelerate the achievement of goals while the patients are undergoing DM therapy [11-13].

Based on these findings, there is a need to develop and validate a prediction score to assess the risk of hypoglycemia in ambulatory T2DM patients. Such a tool would assist health workers in identifying and prioritizing patients who may benefit from additional supportive services. Therefore, this study aims to (1) identify risk factors associated with hypoglycemia in T2DM patients, (2) convert these risk factors into a practical scoring system, (3) classify risk levels as low, moderate, or high, and (4) evaluate the relationship between the risk categories and the actual incidence of hypoglycemia. The ultimate goal is to provide a simple and effective tool for healthcare providers to assess hypoglycemia risk and ensure that patients in the moderate and high-risk groups receive appropriate preventive support at home [14-16].

## 2 MATERIAL AND METHODS

### 2.1 Study Design and Setting

This study used a psychometric properties method, including item development, content validity, criterion validity, and construct validity [17-19]. In the item development stage, qualitative methods were used through Focus Group Discussions (FGD) with experts. The results of the FGDs were synthesized into predictors influencing the incidence of hypoglycemia. Known predictors were then validated through panelists' content assessment. Risk factors for hypoglycemia from item development and content validation were tested for criteria and construct validation on T2DM patients. The study design used at this stage was a case-control method to examine the predictors contributing to hypoglycemia according to the characteristics of patients at the study site. Criterion validation was conducted using a predictive validity approach, where a case-control analysis was performed on the risk factors of T2DM patients with a history of severe hypoglycemia (as cases) and patients without a history of severe hypoglycemia (as controls). The results of the multivariate analysis of significant risk factors were stated as valid risk factors [17]. Construct validity was assessed to determine whether the identified risk factors behaved as expected when compared across “known groups,” thereby confirming the tool’s ability to distinguish between patients at different levels of hypoglycemia risk. This validation was conducted by grouping patients who have a number of few, medium, and many risk factors based on the odds ratio value of the validation criteria results. The total odds ratio value of all valid criteria was divided and then grouped into low, medium, and high-risk categories. Analysis was conducted to see the correlation between scale scores and existing measurements. The results were declared construct valid if there was a significant correlation between the variables [17, 18].

This study was conducted in Bali Province, and all participants involved were part of a multicenter group of three hospitals in the province of Bali, located in Denpasar City, Badung district, and Buleleng district. This investigation contributed to the development of an instrument for assessing the risk of hypoglycemia in patients with T2DM. The primary outcome was the determination of risk factor scores, analyzing the correlation of the determined risk scores with the incidence of hypoglycemia in T2DM patients. Furthermore, the presentation of results was based on Strengthening the Reporting of Observational Studies in Epidemiology (STROBE) guidelines for case-control design [20].

### 2.2 Study Participants and Data Collection

Participants consisted of experts in FGDs, practitioners to validate content, and T2DM outpatients to validate criteria and constructs of risk factors. Experts involved in the FGD were endocrinologists, nurses, clinical pharmacists, community pharmacists, and nutritionists. Content validity included five panelists consisting of practitioners at Bali provincial hospitals who have been treating ambulatory T2DM patients with more than five years of experience. The T2DM patients were diagnosed with the condition at three government hospitals in the province of Bali, Indonesia. The inclusion criteria consisted of patients with available data on risk factors identified during item development, including gender data, age (>20 years), Body Mass Index (BMI), duration of DM, last blood glucose profile, ambulatory diabetes medications (oral or parenteral), and presence of comorbidities associated with T2DM.

All of these risk factors were mandatory to be found in patient's medical records as independent variables for data analysis. Exclusion criteria included patients who met the inclusion criteria but declined to participate, those who were not monitored monthly over the past year, and patients with incomplete, inconsistent, or illegible medical records.

The initials were sorted by ICD-10 codes E11 and E16.2 to obtain patients' record numbers, which were taken sequentially according to their database from January 2022 to May 2024 for review [21]. The case group included T2DM patients who were recorded as having a history of hypoglycemia hospitalization and received parenteral fluid therapy in the form of dextrose 10% (D10) in the medical record. Meanwhile, the control group included T2DM patients without a history of hypoglycemia hospitalization in their medical records.

The two groups were retrospectively analyzed to examine the predetermined independent variables and identify which of these were significantly associated with hypoglycemia in T2DM patients.

Medical record data were extracted from three multicenters involving six observers. The extracted data, adhering to the study criteria, were reviewed by the team to ensure completeness, accuracy, and precision. This review step was necessary because the multicenter had not fully implemented electronic medical records, resulting in a significant amount of handwritten notes by practitioners. This situation could lead to data discrepancies, interpretation errors, and misunderstandings regarding the patient's therapy course.

### 2.3 Study Size

In this study, the design was expected to involve a minimum of five experts for FGD and five practitioners for validation. By involving five panelists, the CVR value was declared valid if it was 0.99 from the formula used, which was as follows (1) [22-24]:


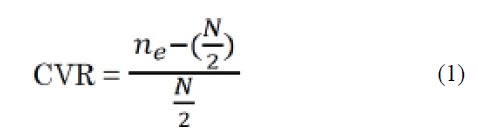


CVR: Content Validity Ratio

ne: Instrument items approved by panelists

N: Total number of panelists assessing

A minimum sample size of 200 T2DM patient records was required for the case-control design to adequately represent the population, with at least 100 patients assigned to both the case and control groups. The minimum sample size formula used was as follows (2) [25, 26]:


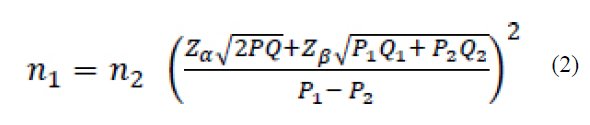


n1: Number of subjects in T2DM patients in the control group

n2: Number of subjects in T2DM patients in the case group

α: Type one error (set at 5%)

β: Type two error (set at 10%)

Zα: Standard alpha value (1.96) for two-way hypothesis

Zβ: Standard beta value (1.28)

P_1_: Proportion of T2DM patients without risk of hypoglycemia

Q_1_: 1 - P_1_

P_2_: Proportion of T2DM patients with a risk of hypoglycemia

Q_2_: 1 - P_2_

P: (P_1_ + P_2_) / 2

Q: 1 - P

Subsequently, data collection was conducted through direct observation during FGDs and content validation with expert and practitioner panelists. Patient data were obtained from a multicenter database.

### 2.4 Study Variables and Potential Bias of Study

The primary variables were divided into dependent and independent categories. The dependent variable included the incidence of hypoglycemia in T2DM patients based on medical records. Meanwhile, the independent variables were gender, age, BMI, DM duration, uncontrolled blood glucose, diabetes medications (oral or parenteral), and T2DM patient's comorbidities. The independent variables were subjected to a multivariate analysis to determine the significant factor that affected hypoglycemia and its interactions between these independent variables.

This investigation had some potential biases, such as not being able to obtain ideal patient characteristics between the case and control groups due to study design limitations that depended on the availability and quality of the data in the medical database. Variables that may have influenced hypoglycemia events in ambulatory T2DM patients but were difficult to assess retrospectively included medication adherence, nutritional intake, the patient's beliefs, and their support system at home [27-29].

### 2.5 Data and Statistical Analysis

FGD data were processed using the free version of the QDA Miner Lite software [30]. The predictors obtained from the FGD were validated using Content Validity Ratio (CVR) analysis, with a minimum CVR value of 0.99 from five panelists. The case-control study data were analyzed through several stages. The first stage of predictors was analyzed through chi-square. A *p*-value <0.25 was considered significant multivariate binary logistic regression analysis. In multivariate analysis, the independent variable was declared significant if the *p*-value was <0.05. The statistically significant factors were calculated using the Odds Ratio (OR) and 95% Confidence Interval (CI) and then converted into a score. For the scoring system, values were rounded to the nearest whole number, with a cutoff of 0.5. The significant risk factors identified in the multivariate analysis were validated for criterion validity through the scoring system [25, 31].

Further analysis was carried out to divide the total score into three parts, namely scores representing low, moderate, and high risk in T2DM patients for experiencing hypoglycemia [17, 19]. The score was determined using the middle value of the total score distance. After score categories were obtained, the analysis continued to determine the relationship and correlation of scores with the incidence of hypoglycemia in T2DM patients [32-34]. This analysis was carried out by reassessing all patient data that met the previous inclusion and exclusion criteria according to score categories that had been determined in the criterion validity test. The scoring system was declared construct valid if there was a significant difference (*p*<0.05) in bivariate statistics (chi-square) and a positive correlation in the Spearman rank test between risk score categories and the incidence of hypoglycemia in T2DM patients.

## 3 RESULTS

This study was part of a larger investigation under the auspices of the ambulatory T2DM medication safety project. Medical records from 1,231 multicenter sites were screened to identify patients who met the study criteria. A total of 320 records were excluded due to incomplete or scattered data, 288 patients were recorded as deceased, and 363 did not meet the inclusion or exclusion criteria. Ultimately, 260 ambulatory T2DM patients were successfully screened and met the study criteria, with their characteristics summarized in Table **1**.

The results of the FGDs with experts found eight predictor groups that could be the cause of hypoglycemia in T2DM patients, including gender, age, DM treatment, specifically insulin and sulfonylurea (SU), uncontrolled DM complications, uncontrolled blood glucose, longer DM duration, non-ideal BMI, and a history of severe hypoglycemia. These predictors were assessed for content validity by clinicians practicing at the multicenter hospital of the study site. The results of content validation are shown in Table **2**. Content validation results eliminated gender predictors. Meanwhile, the DM complication predictor was only valid for diabetic neuropathy and Chronic Kidney Diseases (CKDs). Finally, all predictors were subjected to bivariate analysis (Table **3**) to assess differences between medical practitioners' perspectives and the actual conditions of hypoglycemia events. These differences were interpreted as part of the criterion and construct validation process.

Bivariate analysis showed that there were significant differences in the predictors of age, insulin use, SU use, insulin-SU combination use, CKD, diabetic neuropathy, blood glucose profile, DM duration, and history of severe hypoglycemia. Gender and BMI did not differ significantly, but the *p*-value was <0.25. Thereby, this predictor was included in the multivariate analysis. DM complications in the form of CVD, DM retinopathy, DMDF, DM gastropathy, hypertension, and dyslipidemia were not included in the multivariate analysis. They did not contribute significantly as predictors of the cause of hypoglycemia in T2DM patients.

According to Table **4**, the results of the multivariate logistic regression analysis showed that there were eight risk factors that most strongly influenced the incidence of hypoglycemia. Gender, age, and BMI were excluded as predictors because they did not have a strong influence on the incidence of hypoglycemia in T2DM patients. Additionally, T2DM patients with uncontrolled blood sugar had a fivefold increased risk of hypoglycemia (CI 95%: 2.294-11.746). Those using a combination of insulin and sulfonylureas (SU) and having more than 5 years of diabetes duration had a fourfold increased risk (CI 95%: 1.580-12.817 and CI 95%: 2.318-8.268).

The use of insulin therapy alone was associated with a threefold increased risk of developing hypoglycemia (CI 95%: 1.602-5.240). T2DM patients using Sulfonylureas (SU) who also have Chronic Kidney Disease (CKD), diabetic neuropathy, and a history of hypoglycemia were found to have up to twice the risk of hypoglycemia (CI 95%: 1.122-4.774, CI 95%: 1.262-4.543, CI 95%: 1.145-3.333, and CI 95%: 1.142-3.276). These significant risk factors identified in the multivariate analysis were considered valid for criterion validity [17-19, 35].

The OR results from multivariate analysis were converted into scores. The risk profile for hypoglycemia was divided into three categories, namely low, moderate, and high risk. The total score of the eight risk factors was 24. Thus, the categories were assigned scores as follows: 0-8 for low risk, 9-16 for moderate risk, and 17-24 for high risk. Score characteristics of T2DM patients after reassessment with scores are presented in Fig. (**1**) and Table **5**. Fig. (**1**) shows a visualization of the distribution of scores from T2DM patients using the scoring system. The hypothesis is that patients without a history of hypoglycemia are more likely to fall into the low-risk score category with fewer risk factors.

In contrast, those with a history of hypoglycemia were more likely to be in the high-risk score category with the highest risk factors. Table **5** shows detailed characteristics of T2DM patients with their risk factors. Patients in the low-risk category were seen to have 2 out of 8 risk factors with an average score of 5 ± 1. Patients in the moderate-risk category had an average of 5 out of 8 risk factors with an average score of 13 ± 1. Patients in the high-risk category had an average of 7 out of 8 risk factors with an average score of 19 ± 1. The results of the correlation analysis of this scoring system are shown in Table **6**.

Table **6** shows the results of statistical tests on the scoring system, which found a positive linear relationship between the total score and risk category. The greater the score value, the higher the risk of hypoglycemia (*p*<0.05). The results state that the predictors and scoring system can be used to assess the risk of hypoglycemia in ambulatory T2DM patients.

## 4 DISCUSSION

Eight risk factors were found to be valid in terms of criterion validity, including uncontrolled blood glucose, insulin use, sulfonylurea (SU) use, insulin-SU combination therapy, diabetes duration of more than 5 years, Chronic Kidney Disease (CKD), diabetic neuropathy, and a history of hypoglycemia. The use of insulin and sulfonylureas has been widely reported to significantly increase the risk of hypoglycemia [19, 36, 37]. The most common cause of this incident was found to be a significant increase in blood insulin levels without adequate nutritional intake [22-25]. A history of hypoglycemia was identified as a significant contributor to the risk of experiencing future hypoglycemic episodes [38]. While this phenomenon cannot be definitively confirmed, several studies suggest that recurrent hypoglycemia may impair the brain’s ability to sense and respond to subsequent hypoglycemic events. DM duration and uncontrolled blood glucose were found to be contributing factors because they are often associated with diabetes complications. The longer patients live with unstable glucose control, the greater their risk of developing complications, experiencing medication errors, and encountering adverse drug reactions (ADRs) [39-42].

CKD and diabetic neuropathy are the most dominant DM complications that influence the incidence of hypoglycemia. CKD often affects a patient's physiological function and changes the pharmacokinetic and pharmacodynamic parameters of antidiabetic drugs [43, 44].

As a result, failure to adjust medication doses and lack of self-monitoring increase the risk of hypoglycemia in T2DM patients with CKD. Diabetic neuropathy is frequently associated with reduced nerve sensitivity to low blood glucose levels, further contributing to hypoglycemia risk [45-49].

Glucose-sensing neurons in the ventromedial hypothalamus nucleus (VMN) are crucial for detecting hypoglycemia. Moreover, the presence of chronic neuropathy can affect VMN activity [45-48].

Notably, age and BMI emerged as significant risk factors. The findings from the FGDs and content validation suggest that these two factors may influence the incidence of hypoglycemia. Several studies have shown that older age and non-ideal BMI are associated with an increased risk of hypoglycemia in T2DM patients [50-69]. Both elderly and adult patients, as well as those with ideal and non-ideal BMIs, were equally represented in both the hypoglycemia (case) and non-hypoglycemia (control) groups. Several risk factors that influence hypoglycemia in T2DM patients with varying results include gender, age, and BMI because these predictors depend on the unique characteristics of patients, such as genetics and background, which play an essential role in the variation of hypoglycemia incidence in T2DM patients [70-72].

The scoring system developed to assess hypoglycemia risk in T2DM patients demonstrated a strong positive correlation between the total score and the incidence of hypoglycemia in patients. The correlation coefficient was found to be very strong, indicating that the scoring model effectively predicts the future risk of hypoglycemia. This simulation can now be used to map the hypoglycemia risk profile of T2DM patients who are managing their condition at home. Ambulatory T2DM patients are actually at risk of developing adverse drug reactions, predominantly hypoglycemia, when undergoing diabetes therapy [57, 58]. Mapping the risk of ambulatory T2DM patients regarding the potential for hypoglycemia at home will be very helpful for physicians, clinical pharmacists, nurses, and nutritionists when educating patients [73-77]. Health workers can use this information to prioritize patients for more intensive preventive and promotive services based on their hypoglycemia risk category.

## CONCLUSION

In conclusion, risk factors influencing the incidence of hypoglycemia in T2DM patients included uncontrolled blood glucose, insulin use, SU use, use of insulin-SU as a combination therapy, DM duration of 5> years, CKD, diabetic neuropathy, and a history of hypoglycemia. The total prediction score for all these risk factors was divided into three categories, namely low, moderate, and high risk of hypoglycemia cases. This risk factor and score prediction model can be used by health workers in clinical practice to predict the hypoglycemia risk of ambulatory T2DM patients because it meets content, criterion, and construct validity.

## STUDY LIMITATIONS

This study has several limitations, including the fact that predictors were measured based on medical records in a multicenter setting, and the possible time-varying effects of the predictors could not be reported. Another limitation was that patients with T2DM were enrolled in three multicenters in Bali Province, Indonesia. Therefore, the results may not be generalized to other Asian populations because of different genetic backgrounds and healthcare systems.

## RECOMMENDATION

Based on the findings of this study, the following key recommendations are made:

1. The findings in this study can be useful as a reference in screening the risk of hypoglycemia in ambulatory T2DM patients. The results of this study met all the requirements for psychometric properties testing.
2. Health workers are encouraged to screen patients who are discharged on antidiabetic medications for the risk of hypoglycemia, as this can help identify those in need of additional support.
3. Patients with T2DM must be informed about their disease along with self-management strategies to avoid the risk of hypoglycemia that may lead to hospitalization.
4. Continuous promotive, preventive, and curative efforts for ambulatory T2DM patients are needed to control modifiable risk factors with the aim of avoiding the risk of hypoglycemia.

## Figures and Tables

**Fig. (1):**
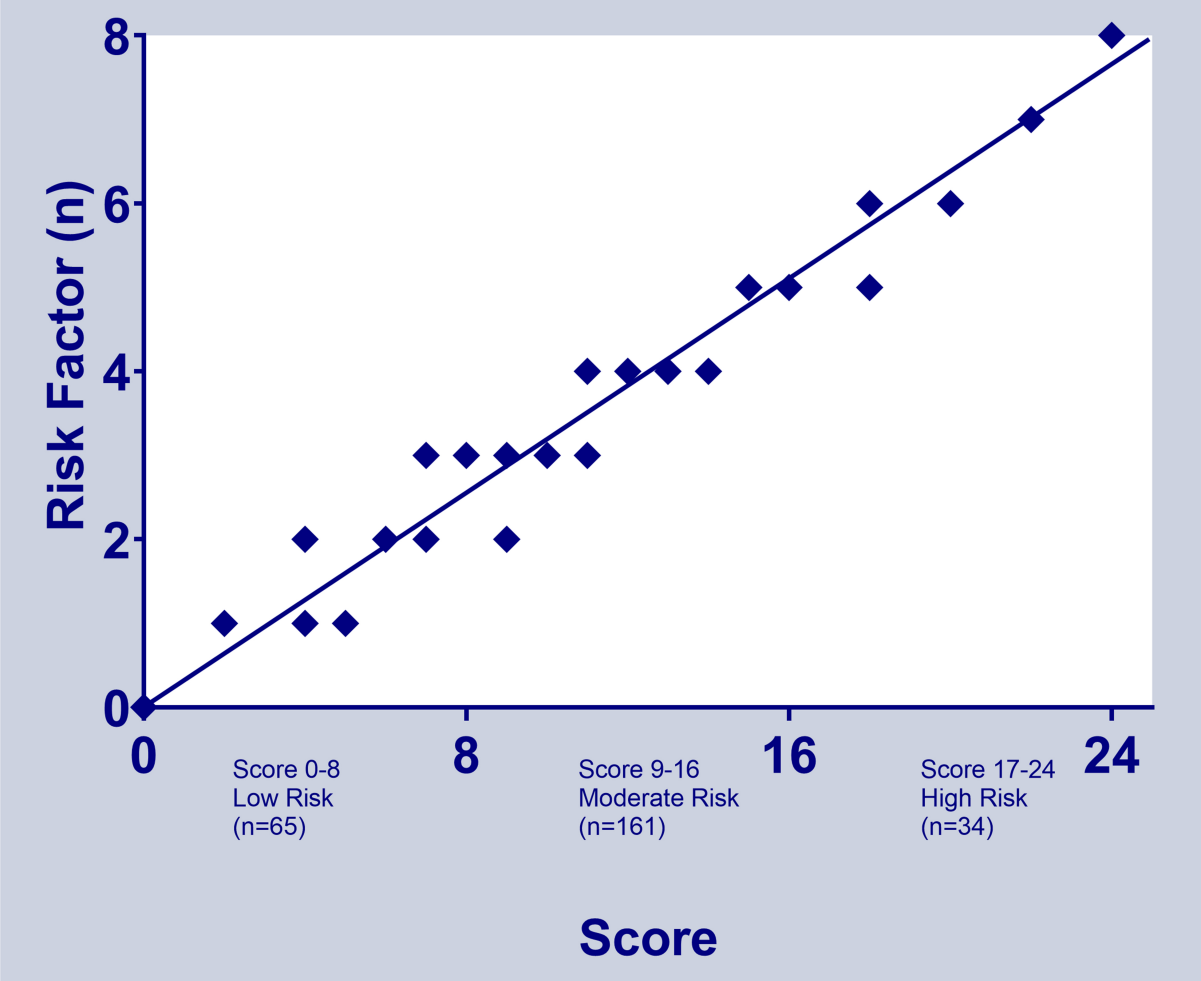
Visual clustering of hypoglycemia risk scores in T2DM patients.

**Table 1:** Characteristics of type 2 diabetes mellitus (T2DM) patients.

**S. No.**	**Characteristic**	**Value (n= 260)**
1	**Gender**	-
	Male (n %)	141(54.23)
	Female (n %)	119 (45.77)
2	**Ages (Years)**	-
	Average ± SD	57.26 ± 12.65
	20-30	5 (1.92)
	31-40	21 (8.08)
	41-50	55 (21.15)
	51-60	77 (29.62)
	>60	102 (39.23)
3	**BMI**	-
	Average ± SD	25.85 ± 4.09
	Under Weight (≤18 kg/m^2^); (n %)	7 (2.69)
	Normal (19-25 kg/m^2^); (n %)	96 (36.92)
	Overweight (26-30 kg/m^2^); (n %)	123 (47.31)
	Obese (>30 kg/m^2^); (n %)	34 (13.08)
4	**T2DM Duration **(Years)	-
	Average ± SD	7.04 ± 3.82
	0-5	67 (12.31)
	6-10	161 (25.77)
	>10	32 (61.92)
5	**Last Blood Glucose Profile**	-
	HbA1c (Average); (%)	9.59 ± 2.97
	Fasting glucose (Average); (mg/dL)	206 ± 85
	Prandial glucose (Average); (mg/dL)	250 ± 92
	Random glucose (Average); (mg/dL)	275 ± 142
	Uncontrolled (n %) (HbA1c >7%, fasting >126mg, prandial & random >200mg/dL)	220 (84.62)
	Controlled (n %) (HbA1c <7%, fasting <126 mg/dL, prandial & random <200mg/dL)	40 (15.38)
6	**T2DM Medication**	-
	Insulin User (n %)	107 (41.15)
	Sulfonylurea User (n %)	48 (18.46)
	Metformin (n %)	132 (50.77)
	DPP-4 Inhibitor (n %)	14 (5.38)
	Alpha Glucosidase Inhibitor (n %)	2 (0.77)
	SGLT2 Inhibitor (n %)	6 (2.31)
7	**Comorbidity and Complication**	-
	CKD (n %)	63 (24.23)
	Neuropathy DM (n %)	101 (38.85)
	Cardiovascular Diseases (n %)	153 (58.85)
	Retinopathy DM (n %)	6 (2.31)
	Diabetic Foot (n %)	48 (18.46)
	Gastropathy DM (n %)	35 (13.46)
	Hypertension (n %)	119 (45.77)
	Dyslipidemia (n %)	58 (22.31)

**Abbreviations: **n: number; SU: sulfonylurea; DM: diabetes mellitus; CKD: chronic kidney diseases; CVD: cardiovascular diseases; DMDF: diabetes mellitus diabetic foot; BMI: body mass index.

**Table 2:** Demographic and clinical characteristics of the study participants in whole and stratified by microvascular complications.

**S. No.**	**Risk Factors Compiled from FGDs with Expert Clinicians**	**Content Assessment Panelists** (**Clinicians Practicing in Health Services**) **n=5**					**CVR**	**Minimum CVR Value (0.99)**
		**1**	**2**	**3**	**4**	**5**		
1	Gender (male)	DA	DA	A	DA	DA	-0.6	Invalid
2	Ages (older)	A	A	A	A	A	1.0	Valid
	a. Insulin User	A	A	A	A	A	1.0	Valid
	b. SU User	A	A	A	A	A	1.0	Valid
	c. Combination Insulin and SU	A	A	A	A	A	1.0	Valid
3	**Having Comorbidity or Complication**							
	a. CKD	A	A	A	A	A	1.0	Valid
	b. Diabetic Neuropathy	A	A	A	A	A	1.0	Valid
	c. CVD	DA	A	A	A	DA	0.2	Invalid
	d. DM Retinopathy	DA	DA	DA	DA	A	-0.6	Invalid
	e. DMDF	DA	DA	DA	DA	A	-0.6	Invalid
	f. DM Gastropathy	DA	DA	DA	DA	A	-0.6	Invalid
	g. Hypertension	A	A	DA	DA	A	0.2	Invalid
	h. Dyslipidemia	DA	A	A	DA	A	0.2	Invalid
4	Uncontrolled blood glucose	A	A	A	A	A	1.0	Valid
5	DM Duration > 5 years	A	A	A	A	A	1.0	Valid
6	Non-ideal BMI (over/under weight)	A	A	A	A	A	1.0	Valid
7	Have a history of severe hypoglycemia.	A	A	A	A	A	1.0	Valid

**Abbreviations: ** n: number; SU: Sulfonylurea; DM: Diabetes Mellitus; CKD: Chronic Kidney Diseases; CVD: Cardiovascular Diseases; DMDF: Diabetes Mellitus Diabetic Foot; BMI: body Mass Index; CVR: Content Validity Ratio; A: Panelists Agreed with the Predictors; and DA: Panelists Disagreed with the Predictors. Severe Hypoglycemia: BG ≤ 30 mg/dL; uncontrolled BG: HbA1c >7%, FG >126 mg, PG >200 mg, RG >200 mg.

**Table 3:** Analysis of risk factors associated with hypoglycemia.

**Variable**	**Hypoglycemia Case (n=138)**	**Non-Hypoglycemia Control (n=122)**	***p* Value (χ2)**
**Gender**			
Male (n %)	70 (50.72)	71 (58.20)	0.227
Female (n %)	68 (49.28)	51 (41.80)	
**Ages (years)**			
Elderly (≥ 65); (n %)	67 (48.55)	41 (33.61)	0.015*
Adult (20< ages <65); (n %)	71 (51.45)	81 (66.39)	
**Medication that often causes Hypoglycemia Events**			
Insulin User (all types); (n %)	66 (47.83)	41 (33.61)	0.001*
SU (n %)	27 (19.57)	21 (17.21)	
Insulin and SU in combination (n %)	15 (10.87)	6 (4.92)	
**CKD**			
Yes (n %)	45 (32.61)	18 (14.75)	0.001*
No (n %)	93 (67.39)	104 (85.25)	
**Diabetic Neuropathy**			
Yes (n %)	65 (47.10)	36 (29.51)	0.004*
No (n %)	73 (52.90)	86 (70.49)	
**CVD**			
Yes (n %)	3 (2.17)	3 (2.46)	0.879
No (n %)	135 (97.83)	119 (97.54)	
**DM Retinopathy**			
Yes (n %)	19 (13.77)	16 (13.11)	0.878
No (n %)	119 (86.23)	106 (86.89)	
**DMDF**			
Yes (n %)	26 (18.84)	22 (18.03)	0.867
No (n %)	112 (81.16)	100 (81.97)	
**DM Gastropathy**			
Yes (n %)	84 (60.87)	69 (56.56)	0.481
No (n %)	54 (39.13)	53 (43.44)	
**Hypertension**			
Yes (n %)	66 (47.83)	53 (43.44)	0.479
No (n %)	72 (52.17)	69 (56.56)	
**Dyslipidemia**			
Yes (n %)	31 (22.46)	27 (22.13)	0.949
No (n %)	107 (77.54)	95 (77.87)	
**Blood Glucose**			
Uncontrolled (n %)	129 (93.48)	91 (74.59)	0.001*
Controlled (n %)	9 (6.52)	31 (25.41)	
**DM Duration (years)**			
≥5 (n %)	118 (85.51)	75 (61.48)	0.001*
<5 (n %)	20 (14.49)	47 (38.52)	
**BMI (kg/m^2^)**			
Over/Under Weight (n %)	94 (68.12)	70 (57.38)	0.073
Normal Weight (n %)	44 (31.88)	52 (42.62)	
**History Severe Hypoglycemia**			
Yes (n %)	79 (57.25)	43 (35.25)	0.001*
No (n %)	59 (41.75)	79 (64.75)	

**Abbreviations: **n: number; SU: Sulfonylurea; DM: Diabetes Mellitus; CKD: Chronic Kidney Diseases; CVD: cardiovascular diseases; DMDF: diabetes mellitus diabetic foot; BMI: body mass index; χ2: Chi-Square analysis; *:statistically significant; *p*-value ≤0.25: Continues to multivariate analysis; severe hypoglycemia: BG ≤ 30 mg/dL; uncontrolled BG: HbA1c >7%, FG >126 mg, PG >200 mg, RG >200 mg.

**Table 4:** Criterion validity of risk factors associated with hypoglycemia.

**S. No.**	**Independent Variable**	***p* Value**	**OR (CI95%)**	**Score**	**Validity (*p*<0.05)**
1	Gender (male)	0.086	0.621 (0.360-1.071)	ND	Invalid
2	Ages (elderly)	0.221	1.421 (0.810-2.494)	ND	Invalid
3	Insulin user (all types)	0.001*	2.898 (1.602-5.240)*	3	Valid
4	SU user	0.023*	2.314 (1.122-4.774)*	2	Valid
5	Insulin and SU in combination	0.005*	4.500 (1.580-12.817)*	4	Valid
6	CKD	0.008*	2.394 (1.262-4.543)*	2	Valid
7	Diabetic Neuropathy	0.014*	1.954 (1.145-3.333)*	2	Valid
8	BMI (over/under weight)	0.202	1.445 (0.821-2.543)	ND	Invalid
9	Uncontrolled blood glucose	0.001*	5.191 (2.294-11.746)*	5	Valid
10	DM Duration (> 5 years)	0.001*	4.378 (2.318-8.268)*	4	Valid
11	Having a history of severe hypoglycemia	0.014*	1.934 (1.142-3.276)*	2	Valid

**Abbreviations: **ND: not determined; *p*-value <0.05 is statistically significant; *: statistically significant in multivariate analysis; OR: Odds Ratio; CI: Confident Interval; SU: Sulfonylurea; DM: Diabetes Mellitus; CKD: Chronic Kidney Diseases; CVD: Cardiovascular Diseases; DMDF: Diabetes Mellitus Diabetic Foot; and BMI: Body Mass Index. Severe hypoglycemia: BG ≤ 30 mg/dL; uncontrolled BG: HbA1c >7%, FG >126 mg, PG >200 mg, RG >200 mg.

**Table 5:** Scoring characteristics for construct validity in T2DM patients with risk factors for hypoglycemia.

**S. No.**	**Independent Variable**	**Low Risk (Score 0-8) n= 65 Average Score ± SD**	**Moderate Risk (Score 9-16) n= 161 Average Score ± SD**	**High Risk (Score 17-24) n=34 Average Score ± SD**
1	Having a history of severe	0 ± 0	1 ± 1	1 ± 1
2	Insulin user (all types)	0 ± 0	2 ± 1	3 ± 0
3	SU user	0 ± 1	0 ± 1	1 ± 1
4	Insulin and SU in combination	0 ± 0	0 ± 1	2 ± 2
5	CKD	0 ± 0	0 ± 1	1 ± 1
6	Diabetic neuropathy	0 ± 1	1 ± 1	2 ± 1
7	Uncontrolled BG	2 ± 3	5 ± 1	5 ± 0
8	DM duration (> 5 years)	1 ± 2	3 ± 1	4 ± 1
**Total average score ± SD**		5 ± 1	13 ± 1	19 ± 1

**Abbreviations: ** n: number; SU: Sulfonylurea; DM: Diabetes Mellitus; CKD: Chronic Kidney Diseases; SD: Standard Deviation; BG: Blood Glucose; FG: Fasting Glucose; PG: Prandial Glucose; RG: Random Glucose. Severe hypoglycemia: BG ≤ 30 mg/dL; uncontrolled BG: HbA1c >7%, FG >126 mg, PG >200 mg, RG >200 mg.

**Table 6:** Demographic and clinical characteristics of the study participants in whole and stratified by microvascular complications.

**Hypoglycemia Event**	**Hypoglycemia Risk Category**			***p* Value (χ2)**	**Spearman Correlation**	**Validity (*p*<0.05)**
	**Low Risk (Score 0-8) n= 65**	**Moderate Risk (Score 9-16) n= 161**	**High Risk (Score 17-24) n=34**			
Yes (n %)	15 (23.08)	94 (58.39)	29 (85.29)	0.001*	*p*: 0.001* r^2^: +0.959	Valid
No (n %)	50 (76.92)	67 (41.61)	5 (14.71)			

**Abbreviations: ** n: number; χ2: Chi-Square analysis; *:statistically significant; r^2^: correlation coefficient.

## Data Availability

Not applicable.

## LIST OF ABBREVIATIONS

ADRs: Adverse Drug Reactions
BG: Blood Glucose
BMI: Body Mass Index
CKD: Chronic Kidney Disease
CVD: Cardiovascular Disease
CVR: Content Validity Ratio
DM: Diabetes Mellitus
DMDF: Diabetes Mellitus with Diabetic Foot
FG: Fasting Glucose
PG: Prandial Glucose
RG: Random Glucose
SD: Standard Deviation
SU: Sulfonylurea
T2DM: Type 2 Diabetes Mellitus Patient

## STANDARDS OF REPORTING

STROBE guidelines were followed.
